# Factors associated with COVID-19 severity among Brazilian pregnant adolescents: a population-based study*

**DOI:** 10.1590/1518-8345.6162.3654

**Published:** 2022-09-20

**Authors:** Brenda Katheryne Duarte, Ana Beatriz Henrique Parenti, Milena Temer Jamas, Hélio Rubens De Carvalho Nunes, Cristina Maria Garcia De Lima Parada

**Affiliations:** 1 Universidade Estadual Paulista “Júlio de Mesquita Filho”, Faculdade de Medicina de Botucatu, Botucatu, SP, Brasil.

**Keywords:** Pregnancy, Pregnancy in Adolescence, Adolescence, COVID-19, Pandemic, Intensive Care Units, Gravidez, Gravidez na Adolescência, Adolescência, COVID-19, Pandemia, Unidades de Terapia Intensiva, Embarazo, Embarazo en Adolescencia, Adolescencia, COVID-19, Pandemia, Unidades de Cuidados Intensivos

## Abstract

**Objective:**

to identify the factors associated with need for intensive care unit admission of Brazilian pregnant adolescents with COVID-19.

**Method:**

population-based non-concurrent cohort study using secondary databases. Brazilian pregnant adolescents who had laboratory confirmation of SARS-CoV-2 by RT-PCR, between March 14, 2020 and April 11, 2021 were included in the study. Statistical analysis using the Poisson multiple regression model, estimating the relative risk and respective 95% confidence intervals, with values of *p* <0.05 considered significant.

**Results:**

in total, 282 pregnant women were included in the study, with median age of 17 years, most with brown skin, in the third trimester of pregnancy, and living in urban or peri-urban areas. The intensive care unit admission rate was 14.5%, associated with living in the Southeast region of Brazil (RR=5.03, 95%CI=1.78-14.24, *p*=0.002), oxygen saturation below 95% (RR=2.62, 95%CI=1.17-5.87, *p*=0.019), and having some comorbidity (RR=2.05, 95%CI=1.01-4.16, *p*=0.047).

**Conclusion:**

the intensive care unit admission rate was high among Brazilian pregnant adolescents and was associated with living in the Southeast region of Brazil, having some comorbidity and/or presenting low oxygen saturation.

## Highlights

(1) The ICU admission rate of pregnant adolescents was high: 14.5%.

(2) Low oxygen saturation was a predictor of COVID-19 severity.

(3) Living in the Southeast region in Brazil increased the risk of ICU admission by five times.

(4) Having some comorbidity increased the risk of ICU admission by two times.

## Introduction

The COVID-19 pandemic has caused different impacts on the health of populations. Studies show that children and adolescents develop mild symptoms of the disease, with infrequent admission, intensive care, and oxygen support, and these cases are related to the presence of prior diseases. The hypotheses for a milder disease response in this population group include the strength of the innate immune response, a higher proportion of immunological factors to fight the virus, a lower prevalence of associated comorbidities, close proximity to the Coronavirus family in this age group, and increased colonization of the mucosa by other viruses and bacteria, which could limit colonization by SARS-CoV-2^1^. 

However, when an adolescent becomes pregnant, the progress of COVID-19 tends to be more serious, since the gestational period is characterized by hormonal changes, decreased lung capacity due to the uterus during pregnancy, and a suppressed immune system - aspects that make women more vulnerable to viral infections and related complications^2^ ^-^ ^5^.

A systematic review conducted in the first months of the pandemic showed that pregnant women with COVID-19 have rapidly progressive complications, sometimes associated with comorbidities, leading to a high rate of cesarean deliveries, justified by the worsening of the non-reassuring maternal or fetal status, secondary to worsening of the woman’s clinical status^6^.

Studies report fever, cough, pneumonia, myalgia, fatigue, abdominal pain, and diarrhea as the most recurrent symptoms in pregnant women with COVID-19, and gestational diabetes, hypothyroidism and hypertension as the most commonly associated comorbidities^1^. The outcomes found include higher rates of miscarriage, thyroid disease, coagulopathies, preeclampsia, eclampsia, HELLP (hemolysis, elevated liver enzymes, low platelet count) syndrome, cesarean delivery, preterm birth, need for intensive care unit (ICU) admission, endometritis and/or puerperal sepsis, multiple organ failure, fetal distress and/or low birth weight, pneumonia, and other severe neonatal conditions, asphyxia, and maternal and perinatal death. Then, pregnant women infected with the new Coronavirus have an increased risk of adverse outcomes when compared to the general population^1^ ^-^ ^2^ ^,^ ^7^ ^-^ ^10^.

The maternal mortality ratio estimated for Brazil was 73.8 (2020) and 107.8 (2021) deaths per 100,000 live births, partially resulting from the COVID-19 pandemic^11^, since the lethality rate of the disease among pregnant and postpartum women was 11.7%, while in the general population, 2.8%^12^.

Considering the important effect of COVID-19 on pregnant women and the need for a better understanding of its evolution in different populations and that, so far, no systematic review is available about the evolution of the disease in pregnant adolescents, this study aims to identify the factors associated with need for intensive care unit admission in Brazilian pregnant adolescents with COVID-19. The study hypothesis is that unfavorable sociodemographic conditions, the presence of comorbidities, and the number of clinical signs and symptoms are associated with the need for ICU admission in the studied group.

## Method

### Study design

This is a population-based non-concurrent cohort study.

### Population and sample

This study included Brazilian pregnant adolescents aged 10 to 19 years reported in SIVEP-Gripe from March 14, 2020 to April 11, 2021, totaling 46 epidemiological weeks: weeks 11 to 53 in 2020 and 1 to 4 in 2021, with laboratory confirmation of SARS-CoV-2 by RT-PCR and records of disease progress (ICU admission: yes, no). Data collection was performed in April 2021, excluding adolescents who had no response records in the study variables.

**Figure 1:**
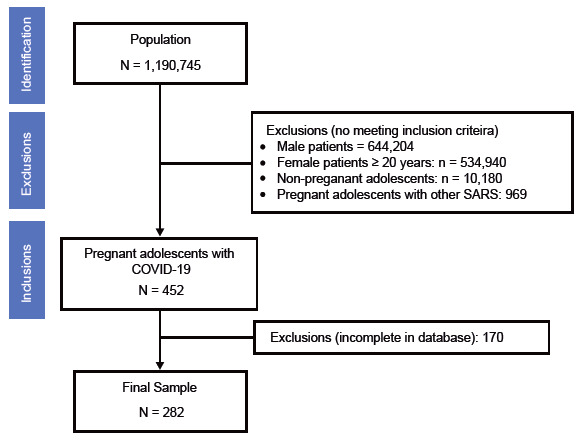
Flow diagram adaptation showing the study sample selection, based on data from the influenza surveillance information system (SIVEP-Gripe). Brazil, 2020-2021.

During the study period, 1,190,745 cases of COVID-19 were reported in Brazil. For this analysis, the sample was selected using the following exclusion criteria: male patients (644,204), female patients ≥20 years (534,940), non-pregnant adolescents (10,180), pregnant adolescents with other severe acute respiratory syndromes (SARS) (969), resulting in 452 cases. Then, cases with incomplete data in the database (170) were excluded, resulting in a final sample of 282 cases (Figure 1). 

### Data collection

This study used the secondary database of the influenza surveillance information system (SIVEP-Gripe), which contains information of all cases of severe acute respiratory syndrome in Brazil, including cases of COVID-19 (database INFLUD-12- 04-2021), offered by the Ministry of Health of Brazil (https://opendatasus.saude.gov.br/dataset/bd-srag-2020). 

### Study variables

The outcome variable was the need for ICU admission (yes, no) of pregnant adolescents with confirmed diagnosis of COVID-19. The independent variables were:

- **Sociodemographic aspects:** age in years of pregnant adolescents (11-14, 15-19), skin color/race (yellow, white, brown, black, indigenous), education (illiterate, elementary school - first years, elementary school - late years, high school, higher education, and ignored), region of residence (North, Northeast, Central West, Southeast, South), area of residence (urban or peri-urban, rural), and gestational age in trimester (first, second, third). Education and area of residence were excluded from the bivariate analysis due to a high number of ignored cases, so the pregnant women were analyzed in a single age group.
- **Clinical signs and symptoms (yes, no):** fever, cough, dyspnea, respiratory distress, oxygen saturation below 95%, diarrhea, vomiting, abdominal pain, fatigue, anosmia, and ageusia. Other signs and symptoms (field “others” of the compulsory notification form): odynophagia, tachycardia, nasal congestion, inappetence, skin rash, edema, jaundice, chest pain, vertigo, back pain, nausea, chills, asthenia, myalgia, runny nose, seizures, dysuria, headache, and malaise. Considering that any event must be reported in the SIVEP-Gripe system, this variable was analyzed in a dichotomous manner, with ignored cases (blank) considered as “no” clinical signs and symptoms.
- **Presence of comorbidities (yes, no):** cardiovascular, hematological, neurological, renal, hepatic, lung disease, asthma, diabetes mellitus/gestational diabetes, immunosuppression, and obesity. Other comorbidities (field “others” of the compulsory notification form): chronic arterial hypertension, gestational hypertension/preeclampsia, thyroid disease, and alcohol/drug use. Considering that any event must be reported in the SIVEP-Gripe system, this variable was analyzed in a dichotomous manner, with ignored cases (blank) considered as “no” comorbidity.

### Data analysis

First, data were analyzed by descriptive statistics. Then, the association between the independent variables and the need for ICU admission of pregnant adolescents due to COVID-19 complications was identified using the Mann-Whitney, Chi-square or Fisher’s Exact tests. Signs and symptoms with prevalence above 5.0% was included in the bivariate analysis. Then, the variables that most influenced the outcome (*p*<0.20) in the bivariate analysis were inserted in the Poisson multiple regression model and, as a result, relative risks (RR) and the respective confidence intervals of 95% (95%CI) were estimated. In the multiple regression model, associations were considered statistically significant with *p* <0.05. The analyses were performed using SPSS v.21.0.

### Ethical aspects

The ethical aspects were observed, according to Resolution of the National Health Council n° 510, of April 7, 2016, sole paragraph, which states that research using publicly available information will not be registered or evaluated by the Research Ethics Committee/National Research Ethics Commission (CEP/CONEP) system, item II, pursuant to Law 12527, of November 18, 2011^13^.

Then, as the database used in this study can be accessed by the general public, it did not contain the names of participants or any other information allowing individual identification of the participants; therefore, anonymity was guaranteed and this study did not have to be submitted to the Research Ethics Committee for evaluation.

## Results

Among all 282 pregnant adolescents included in this study, the median age was 17 (11-19) years; most of them had brown skin color (68.8%), were in the third trimester of pregnancy (64.6%), and lived in urban or peri-urban areas (83.7%). Also, 37.7% lived in the Northeast region and 24.1% had completed the late years of elementary school; however, the education variable was absent in 45.0% of the cases (Table 1).

**Table 1:** Sociodemographic characteristics and gestational trimester of Brazilian pregnant adolescents with COVID-19 (n=282). Brazil, 2020-2021

Variables		N	%
**Age**			
	11-14	16	5.7
	15-19	266	94.3
**Skin color/Race**			
	White	66	23.4
	Brown	194	68.8
	Black	11	3.9
	Indigenous	11	3.9
	Yellow	0	0.0
**Education**			
	Higher education	0	0.0
	High school	59	21.0
	Elementary school - late years	68	24.1
	Elementary school - first years	26	9.2
	Illiterate	2	0.7
	Ignored	127	45.0
**Region of residence**			
	North	70	24.8
	Northeast	106	37.7
	Central West	19	6.7
	Southeast	68	24.1
	South	19	6.7
**Area of residence**			
	Urban or peri-urban	236	83.7
	Rural	25	8.9
	Ignored	21	7.4
**Gestational trimester**			
	First	28	9.9
	Second	72	25.5
	Third	182	64.6

Most pregnant adolescents included in the study had fever (61.3%) and cough (57.1%), followed by other less frequent signs and symptoms. Regarding comorbidities, they were present in 42.2% of study participants, the most frequent were asthma (5.0%), lung disease (2.1%), and chronic arterial hypertension (2.1%) (Table 2).

**Table 2:** Signs and symptoms and comorbidities of Brazilian pregnant adolescents with COVID-19 (n=282). Brazil, 2020-2021

Variables		N	%
**Presence of signs and symptoms**			
	Fever	173	61.3
	Cough	161	57.1
	Dyspnea	106	37.6
	Respiratory distress	89	31.6
	Odynophagia	56	19.8
	Anosmia	48	17.0
	Headache	44	15.6
	Oxygen saturation below 95%	39	13.8
	Vomiting	38	13.5
	Diarrhea	32	11.3
	Ageusia	32	11.3
	Fatigue	31	11.0
	Myalgia	28	9.9
	Runny nose	25	8.9
	Abdominal pain	20	7.1
	Other signs and symptoms*	40	14.1
**Presence of comorbidities**		119	42.2
**Types of comorbidities**			
	Asthma	14	5.0
	Lung disease	6	2.1
	Chronic arterial hypertension	6	2.1
	Heart disease	5	1.8
	Hematological disease	5	1.8
	Neurological disease	3	1.1
	Immunosuppression	3	1.1
	Obesity	3	1.1
	Gestational hypertension/preeclampsia	3	1.1
	Liver disease	1	0.4
	Diabetes mellitus/gestational diabetes	1	0.4
	Kidney disease	1	0.4
	Other comorbidities^†^	3	1.0

*Tachycardia, nasal congestion, inappetence, skin rash, edema, jaundice, chest pain, vertigo, back pain, nausea, chills, asthenia, seizure, dysuria, malaise; ^†^Thyroid disorder, alcohol/drug use

Table 3 shows the bivariate associations between the need for ICU admission due to the severity of COVID-19 and sociodemographic variables, signs and symptoms, and comorbidities. The ICU admission rate was 14.5% (n=41). The variables with strongest association in the simple logistic regression (*p*<0.20) were: age (*p*=0.062), region of residence (*p*=0.002), comorbidities (*p*=0.001), cough (*p*=0.010), dyspnea (*p*=0.001), respiratory distress (*p*=0.001), oxygen saturation below 95% (*p*=0.001), myalgia (*p*=0.020), heart disease (*p*=0.023), hematological disease (*p*=0.002), asthma (*p*=0.008), and obesity (*p*=0.056) (Table 3).

**Table 3:** Association between the need for intensive care unit admission and sociodemographic variables, signs and symptoms, and comorbidities (n=282). Brazil, 2020-2021

Variables		Intensive care unit admission		
		No (n=241)	Yes (n=41)	*p*
Age in years [Med (min-max)]*		17(11-19)	18(14-19)	*0.062* ^*†*^
		n (%)	n (%)	*p*
**Skin color/Race**				*0.386* ^*‡*^
	White	52 (21.7)	14 (34.1)	
	Brown	169 (70.1)	25 (61.1)	
	Black	10 (4.1)	1 (2.4)	
	Indigenous	10 (4.1)	1 (2.4)	
	Yellow	0 (0.0)	0 (0.0)	
**Region of residence**				***0.002*** ^*‡*^
	North	65 (27.1)	5 (12.2)	
	Northeast	95 (39.4)	11 (26.8)	
	Central West	15 (6.2)	4 (9.8)	
	Southeast	50 (20.7)	18 (43.9)	
	South	16 (6.6)	3 (7.3)	
**Gestational trimester**				*0.372* ^*‡*^
	First	25 (10.4)	3 (7.3)	
	Second	58 (24.0)	14 (34.1)	
	Third	158 (65.6)	24 (58.6)	
**Presence of signs and symptoms**				
	Fever	146 (60.6)	27 (65.9)	*0.604* ^*§*^
	Cough	130 (53.9)	31 (75.6)	***0.010*** ^*§*^
	Odynophagia	50 (20.7)	6 (14.6)	*0.407* ^*§*^
	Dyspnea	79 (32.8)	27 (65.9)	***0.001*** ^*§*^
	Respiratory distress	66 (27.4)	23 (56.1)	***0.001*** ^*§*^
	Oxygen saturation below 95%	21 (8.7)	18 (43.9)	***0.001*** ^*§*^
	Diarrhea	28 (11.6)	4 (9.8)	*1.000* ^*‡*^
	Vomiting	32 (13.3)	6 (14.6)	*0.806* ^*§*^
	Abdominal pain	18 (7.5)	2 (4.9)	*0.748* ^*‡*^
	Fatigue	26 (10.8)	5 (12.2)	*1.000* ^*§*^
	Anosmia	44 (18.3)	4 (9.8)	*0.260* ^*‡*^
	Ageusia	28 (11.6)	4 (9.8)	*1.000* ^*‡*^
	Myalgia	28 (11.6)	0 (0.0)	***0.020*** ^*‡*^
	Runny nose	23 (9.5)	2 (4.9)	*0.551*‡
	Headache	39 (16.2)	5 (12.2)	*0.645* ^*§*^
**Presence of comorbidities**		92 (38.2)	27 (65.9)	***0.001*** ^*§*^
**Types of comorbidities**				
	Heart disease	2 (0.8)	3 (7.3)	***0.023*** ^*‡*^
	Hematological disease	1 (0.4)	4 (9.8)	***0.002*** ^*‡*^
	Asthma	8 (3.3)	6 (14.6)	***0.008*** ^*§*^
	Neurological disease	3 (1.2)	0 (0.0)	*1.000* ^*‡*^
	Lung disease	5 (2.1)	1 (2.4)	*1.000* ^*‡*^
	Immunosuppression	3 (1.2)	0 (0.0)	*1.000* ^*‡*^
	Obesity	1 (0.4)	2 (4.9)	***0.056*** ^*‡*^
	Gestational hypertension/preeclampsia	2 (0.8)	1 (2.4)	*0.377* ^*‡*^
	Chronic arterial hypertension	5 (2.1)	1 (2.4)	*1.000* ^*‡*^

*Median (minimum value-maximum value); ^†^Mann-Whitney test; ^‡^Fisher’s exact test; ^§^Chi-square test

In Poisson multiple regression analysis, the variables myalgia, hematological disease, and obesity presented very wide CI and were excluded from the analysis. Then, in the final model, the variables independently associated with the need for ICU admission due to COVID-19 were: living in the Southeast region (RR=5.03, 95%CI=1.78-14.24, *p*=0.002); oxygen saturation below 95% (RR=2.62, 95%CI=1.17-5.87, *p*=0.019), and having some comorbidity (RR=2.05, 95%CI=1.01-4.16, *p*=0.047) (Table 4).

**Table 4:** Poisson multiple regression analysis conducted to explain the need for intensive care unit admission. Brazil, 2020-2021

Variables		RR*	95%CI^†^	*p*
**Age**		1.10	(0.87-1.40)	*0.426*
**Region**				
	North	1.00		
	Northeast	2.99	(0.97-9.20)	*0.057*
	Central West	3.13	(0.75-13.08)	*0.118*
	Southeast	5.03	(1.78-14.24)	***0.002*** ^*‡*^
	South	0.92	(0.19-4.44)	*0.920*
**Signs and symptoms**				
	Cough	1.52	(0.72-3.23)	*0.275*
	Dyspnea	1.32	(0.57-3.06)	*0.517*
	Respiratory distress	2.02	(0.99-4.14)	*0.054*
	Oxygen saturation below 95%	2.62	(1.17-5.87)	***0.019*** ^*‡*^
**Comorbidities**		2.05	(1.01-4.16)	***0.047*** ^*‡*^
	Heart disease	1.29	(0.34-4.93)	*0.711*
	Asthma	2.28	(0.78-6.69)	*0.134*

*Relative risk; ^†^Confidence interval; ^‡^Significant result

## Discussion

This study aimed to identify factors associated with COVID-19 severity in Brazilian pregnant adolescents requiring ICU admission, found that living in the Southeast region, having oxygen saturation below 95%, and presenting some comorbidity increased the risk of ICU admission by five times, two and a half times, and two times, respectively. The ICU admission rate was high (14.5%).

A study conducted in Brazil in 2020 assessed pregnant women regardless of age, who needed to be hospitalized due to SARS by COVID-19 and, therefore, with some severity; it found that most women lived in the Southeast region of the country, and the authors associate this finding with the fact that the first cases of the disease were reported in this region, specifically in the states of São Paulo and Rio de Janeiro^14^.

Regarding the risk of ICU admission according to the region of the country, the North region was selected as a reference, as it has the lowest proportion of hospitalizations. Compared to it, the risk of ICU admission was higher in the Southeast region, a result that should be considered with caution for two main reasons: the large extension of the confidence interval obtained and, due to the large territorial extension of the country, the pandemic evolved with distinct temporality. However, the fact that data collection included cases reported over a long period can minimize this effect.

An epidemiological study assessing the severe forms of COVID-19 and the distribution of ICU beds and lung ventilators in Brazil described four latent mortality profiles, with the health regions presenting the highest death rates located in areas with shortage of ICU beds and ventilators, especially in parts of the Northeast, Southeast, and South regions. The author considers the large geographic dimension of the North region, combined with its low population density, may explain the lack of formation of clusters in the region. In this context, the identification of clusters of total ICUs (Brazilian National Health System - SUS and supplementary health) was below expected, in a similar proportion in the Southeast and North regions, 35.8% and 31.1%, respectively. However, the situation was very different when considering the number of ventilators, as the deficit was 93.3% in the North region and 17% in the Southeast region^15^.

Among the signs and symptoms presented by pregnant adolescents, oxygen saturation below 95% was an independent risk factor for ICU admission, which agrees with the finding of another Brazilian study^14^ which assessed pregnant women with progression to death. An American study explains that in COVID-19, silent hypoxia occurs in the early stages of the disease, so that the patient may not have difficulty breathing. It happens because the air sacs in the lungs collapse rather than being filled with fluid or pus, which results in reduced oxygen levels but still has the ability to expel carbon dioxide (CO2). On the other hand, when symptoms of shortness of breath begin, the disease is already in its moderate to advanced levels^16^. Therefore, this sign must be carefully investigated when the first symptoms of the disease appear, especially because they are related to disease worsening.

In our study, just under half of pregnant adolescents with COVID-19 had some comorbidity, and this condition doubled the risk of needing an ICU admission. Asthma and heart disease were the most frequent, and were the same comorbidities found in other national studies with pregnant women^14^ ^,^ ^17^, who also mentioned other diseases, such as diabetes, hypertension, and obesity. In Canada, a study with adult and pediatric patients diagnosed with COVID-19, with 1.1% of these pregnant women hospitalized in the ICU of 32 hospitals during the first half of 2020, identified the most common comorbidities were chronic arterial hypertension, diabetes, and chronic cardiovascular disease^18^. A systematic review that included more than 190 studies compared pregnant and non-pregnant women and showed the presence of comorbidity is a risk factor for worse outcomes among pregnant women^19^.

The Brazilian Society of Cardiology^20^, in its COVID-19 positioning, pointed out that during pregnancy, hemodynamic overload can aggravate the state of underlying heart diseases as a result of an increase in cardiac output in the beginning of the first trimester, reaching its peak in the third trimester, together with reduced peripheral vascular resistance, with greater magnitude. Regarding the alterations related to the respiratory system, the Brazilian Society of Cardiology observes that an increase in uterine volume causes a progressive decrease in total lung capacity and chest compliance; as a result, an associated asthma condition can lead to a rapid and progressive evolution of pneumonia caused by COVID-19, which can lead to severe respiratory failure. Consequently, pregnant women with heart disease and asthma are at high risk for COVID-19.

The study showed a high prevalence of brown pregnant adolescents, living in urban/peri-urban areas, having completed the late years of elementary school, a similar finding of other studies conducted in different Brazilian states^17^ ^,^ ^21^. Studies conducted in Brazil^21^ ^-^ ^22^ showed that black and brown population were more negatively affected by the pandemic, as well as people of lower social class and education, and these groups showed a stronger need to go out to work, and then, they were infected on the job or public transport, since activities performed outside the home increased the contact with other people, making social isolation difficult and favoring virus transmission^21^. It is evident that health determinants must be at the core of policies addressing COVID-19, since the disease does not affect all the population the same way.

Also, most pregnant adolescents who needed ICU admission were in the third trimester of pregnancy (64.6%), which can be attributed to the physiological changes of this period and be related to complications at delivery, in postpartum, and with the neonate. The same findings were observed in a study conducted in Iran^23^, in which 62.5% of hospitalized women were in the third trimester of pregnancy. Brazilian studies often identified the occurrence of COVID-19 in the third trimester of pregnancy, with this rate ranging from 45.8% to 66.7% of pregnant women^14^ ^,^ ^17^. Of the signs and symptoms observed in national and international studies^14^ ^,^ ^17^ ^,^ ^24^ ^-^ ^25^, pregnant adolescents had a higher frequency of fever and cough. Because these symptoms are common and unspecific, extensive testing of this population is required for notification and early treatment of the disease, especially during the flu season.

Since pregnant adolescents with COVID-19 can progress to severe disease, potentially requiring ICU admission, and that such care still presents weaknesses, it is necessary to organize and prepare professionals from the entire maternal and child health care network to ensure the best care, particularly nurses, for their important role in prenatal, birth and postpartum care, and for acting as key agents of comprehensive care, present at all levels of health care^26^ ^-^ ^27^.

Finally, the study limitations refer to the fact that it was conducted with a secondary database, which makes it impossible to use some variables of low quality, as well as the possibility of under-recording of cases, both limitations related to reduced sample. On the other hand, the fact that it is a national bank in a country with continental dimensions, such as Brazil, is a positive aspect to be highlighted.

## Conclusion

This study identified a higher rate of ICU admission among pregnant adolescents living in the Southeast region who presented oxygen saturation below 95% and prior comorbidities. A high prevalence of comorbidities and ICU admission was found in the study sample. Then, careful prenatal follow-up is required, also for the identification of comorbidities in pregnant adolescents with COVID-19.
